# Nutrient footprint versus EPA + DHA security in land-locked regions—more of local pond farmed, imported marine fish or fish oil capsules?

**DOI:** 10.1038/s41538-023-00224-z

**Published:** 2023-09-09

**Authors:** Koushik Roy, Petr Dvorak, Zdenka Machova, Jan Mraz

**Affiliations:** Faculty of Fisheries and Protection of Waters, South Bohemian Research Center of Aquaculture and Biodiversity of Hydrocenoses, University of South Bohemia in České Budějovice, Na Sádkách 1780, 370 05 Ceske Budejovice, Czech Republic

**Keywords:** Agriculture, Systems biology, Environmental impact

## Abstract

EPA + DHA intake in land-locked central Europe (CE) is barely fulfilled. Imported marine fish/farmed salmonids are likely the backbone of an ailing EPA + DHA security. Supplementing with captured marine fish oil capsules (~0.5 g up to 1.6 g CO_2_-eq. mg EPA + DHA^−1^) could be comparable in GHG emissions with fish consumption itself (~1 g to as low as 0.6 g CO_2_-eq. mg EPA + DHA^−1^). But synergistic benefits of EPA + DHA intake by consuming fish protein need consideration too. Taking semi-intensive pond carp and intensively farmed salmon as models, we analyzed footprint, eco-services, and resource use efficiency perspectives of achieving EPA + DHA security in a CE region. Despite a lower production footprint, pond-farmed fish greatly lag in EPA + DHA supply (carp 101–181 mg 100 g^−1^ < salmon 750–1300 mg 100 g^−1^). It doubles-to-quadruples footprint ‘per mg’ of EPA + DHA: nitrogen (carp 18.3 > salmon 8.7 mg N), phosphorus (carp 6.8 > salmon 1.6 mg P), and climate change (carp 1.84 > salmon 0.8 g CO_2_-eq.). With enhancements in pond carp (>300 mg EPA + DHA 100 g^−1^), these differences may cease to exist. Harnessing EPA + DHA bioaccumulation pathways active in ponds, finishing feeding strategies, and polyculture, the EPA + DHA content in pond fish may be increased. Ecosystem services with EPA + DHA mining from pond food web or high EPA + DHA output-to-input ratio (pond carp 1–200 > RAS salmon 0.75) make ponds an eco-efficient system. As fish consumption in CE must improve, pond-farmed fish would be needed to complement (but not substitute) salmonid/marine fish/oil capsules consumption. Achieving EPA + DHA security with minimum pressure on the environment or global resources.

## Introduction

The importance of long-chain (>C20), omega-3 series, polyunsaturated fatty acids (ω3 LC-PUFAs), especially eicosapentaenoic acid (EPA) and docosahexaenoic acid (DHA), in the human diet is globally recognized^1^. Based on the minimum recommended dose for cardiac health of the general population (250 mg EPA + DHA person^−1^ day^−1^), the minimum global demand for ω3 LC-PUFA can easily be calculated (250 mg day^−1^ × 365 days × 7.8 billion population) to the amounts of over 0.7 million tons (mt); on an ideal consumption basis (500 mg EPA + DHA person^−1^ day^−1^), the demand is even higher (>1.4 mt)^2^. The supply of EPA + DHA for human consumption is presently estimated to be around 420 kilotons (kt) yr^−1^, or 149 mg EPA + DHA per capita daily, representing only 30% of the global demand of 1.4 mt^3^. In other words, just more than half (60%) of the minimum demand of EPA + DHA (0.7 mt) is currently fulfilled. Significant losses also occur due to food waste (219 kt EPA + DHA yr^−1^) and unutilized fisheries by-products (53 kt EPA + DHA yr^−1^)^3^. Sustainable optimization of the global aquatic omega-3 supply chain could narrow the demand-supply gap; up to 630 kt EPA + DHA yr^−1^ could be extracted from the human food chain^4^. Accounting for the increased human population, effects of climate change, and decreasing fish abundances in marine waters, those are likely to reduce ω3 LC-PUFA supply in the future^4^. This stark reality of ω3 LC-PUFA demand and supply gap, which is relevant globally, has been the subject of much analysis^2–4^. By the year 2050, the current EPA + DHA supply (>400 kt yr^−1^) must be doubled (>800 kt yr^−1^)^4^. It is believed the available amount of EPA + DHA in oceans exceeds human demand manifold. Marine microalgae, thraustochytrids, scyphozoan pelagic jellyfish, amphipods, and whelk are being discussed as untapped treasures in oceanic EPA + DHA supply for human food basket^5^. Questions of feasibility or impact of harvesting at such lower mesh sizes in the ocean or upscaling production remain.

To date, EPA/DHA supply from aquatic food (or blue food, https://bluefood.earth/) is globally the most highlighted pathway^6,7^. Scoping for novel alternatives of ω3 LC-PUFAs, such as transgenic camelina/ canola oil, oleaginous yeast, or marine microalgae directly for the human food chain, are being explored^2^. Many of these alternatives are also used in fish feed to reduce the use of marine fish oil^8^. Even among the aquatic foods, the EPA + DHA density (richness) of ‘blue food’ varies depending on the trophic level (e.g., small pelagic fishes versus large predators) or fattiness of fish (carp versus tilapia; salmon versus cod)^6^. So, their ability to nourish nations also depends on the choice of fish consumed. Some farmed fish species are ‘net consumers’ of ω3 LC-PUFAs while some are ‘net producers’^3,4^. Because of the unique omega-out-omega-in conversion ratio (ωCR) each fish species or their farming systems provide^4^, the choice of some blue foods (on human food plate) could have a higher environmental footprint of achieving EPA + DHA security, while others could minimize it. The stoichiometry of edible fish biomass originating from extensive to semi-intensive ponds (e.g., carp) or intensive recirculatory aquaculture system (RAS; e.g., salmonids, percids) would determine the environmental costs of achieving EPA + DHA security in a country. A formal analysis in this regard is so far under-represented in literature and policymaking.

The world is consuming more fish than it ever did (20.2 kg capita^−1^), contributing about 17% of animal protein, reaching over 50% in several countries in Asia and Africa^9^. Most national health advisories suggest consumption of ‘at least’ two portions of fish (preferably oily) per week (200 g portion^−1^)^10^, amounting to ≥20 kg capita^−1^ year^−1^. Many organizations, including in Czechia, do not mention explicitly if fish must be marine or oily; rather say eat a variety of fish^11^. Recent ‘planetary healthy diet’^12^ also suggests the replacement of meat with fish intake, maintaining a balance of ~1:1 (chicken: fish) or ~0.5:1 (red meat: fish). However, some regions are aligned in opposite directions to such idealism. For example, the predominantly land-locked Central Europe (CE) has the lowest population average of EPA + DHA levels in blood, globally classified as “very low”^13^. The omega-3 index of the adult human population is below 2.5–3.5%^13–15^. Fish consumption is low (6–8 kg capita annum; <1 fish portion week^−1^) at alarmingly high terrestrial meat intake (64–83 kg capita annum). The food balance looks ~5:1 for chicken:fish and 9:1 for red meat:fish^16–19^. The EU mortality map attributable to a diet low in omega-3 fatty acids (from fish) shows high-risk zones in ‘land-locked’ CE. The population likely consumes too high amounts of saturated fatty acids. Two solutions could be envisaged for a healthy and eco-friendly solution: (a) avoiding red meats and other sources of saturated fatty acids and (b) downplaying one component (saturated fats) with increased consumption of another component (LC-PUFAs). The present study focuses on the latter solution, as the former solution is being debated^20,21^.

Globally aquaculture is the fastest major food production sector, and already half of the blue food in human food basket are farmed. However, the inland aquaculture in CE has not grown substantially to cover local human food baskets. The majority (~70%) of consumed fishes are of import origin and mostly marine species, both wild and farmed (presented later). If aquaculture is further increased, it would either happen at the expense of dilution of fatty acid levels in the fillet^2,10^ or valorizing alternative sources for EPA + DHA security^3,4,8^. The present study took Czechia, as a representative territory in CE, with known blue foods production pattern (e.g., ~80–90% from ponds and carp), their nutrient footprints (e.g., N, P, CO_2_-equivalent per unit production or consumable weight), slaughterhouse efficiency (e.g., up to 60% edible yield from carp, 60% from salmonids), nutrient density (e.g., ω3 LC-PUFA content per unit edible biomass), societal consumption pattern (fish oil capsules, mostly marine fish, and farmed salmon), and ecosystem services of certain blue food production system (e.g., ponds). With these baselines, the study aimed to synthesize the following information: (a) the current status of EPA + DHA security in the region; (b) greenhouse gas emissions associated with increasing EPA + DHA intake from different sources; (c) comparative farm nutrients effluent, ecosystem services, resource use efficiency between carp and salmon production models for the production of EPA + DHA; (d) EPA + DHA bioavailability and accumulation from pond diets to carp in Central European fishponds—a non-FM/FO method.

## Results

### Current status of EPA + DHA security in the region (Czech scenario)

The balance between farmed and captured fish consumption is almost 1:1. Marine fish dominate in the fish food plate (61.5%). A detailed breakup of EPA + DHA source and supply in Czech fish food plate is given in Table 1. By volume, salmon and carp were the most representative farmed fish in the Czech fish food plate. Canned herring, fish oil capsules, and smoked mackerel are the most potent sources of EPA + DHA (Table 1). The current daily EPA + DHA intake per capita is 217.8 mg, which is 87% of the minimum recommended 250 mg EPA + DHA intake day^−1^ by the National Health Advisory.

**Table 1:** Breakdown of aquatic food sources and EPA + DHA supply patterns in land-locked Czechia (typical for Central Europe).

Rank	Item	mg EPA + DHA content g^−1^	g capita^−1^ day^−1^ consumed (% of total)	mg EPA + DHA capita^−1^ day^−1^ supplied (% of total)
1.	Herring	26.6	2.25 (13.5%)	59.93 (27.5%)
2.	Fish oil capsules	250	0.20 (1.2%)	50.00 (23%)
3.	Mackerel	24.5	1.45 (8.7%)	35.45 (16.3%)
4.	**Salmon**^**a**^	15.1	1.73 (10.4%)	31.41 (14.4%)
5.	Sardine	28.8	0.61 (3.7%)	17.52 (8%)
6.	Trout	21.4	0.30 (1.8%)	6.45 (3%)
7.	**Carp**^**a**^	1.5	4.00 (24%)	6.00 (2.8%)
8.	Tuna	2.5	1.58 (9.5%)	3.94 (1.8%)
9.	Pangasius, tilapia	1.03	1.89 (11.4%)	1.95 (0.9%)
10.	Cod	2.3	0.71 (4.2%)	1.62 (0.7%)
11.	Surimi	2.5	0.44 (2.6%)	1.10 (0.5%)
12.	Hake	0.90	1.00 (6%)	0.90 (0.4%)
13.	Oyster, clam	28.8	0.16 (1%)	0.74 (0.3%)
14.	Shrimp	2.1	0.19 (1.2%)	0.40 (0.2%)
15.	Pike, perch, catfish	2.83	0.14 (0.8%)	0.34 (0.2%)
**Total**			**16.6**^**b**^ **(100%)**	**217.8**^**c**^ **(100%)**

Items are ranked in descending order of EPA + DHA supply on the food plate.

^a^Selected for case study (see Methods).

^b^Corresponds to 6 kg fish consumption capita^−1^ year^−1^.

^c^Fulfilling 87% of minimum recommended daily EPA + DHA intake (250 mg).

Boldfaced items are selected for case study.

**Fig. 1:**
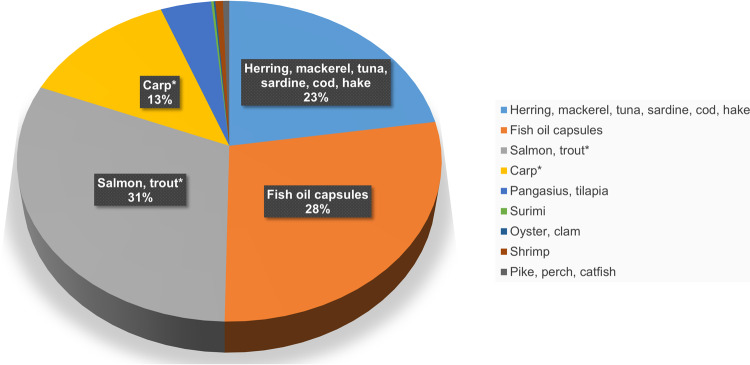
Breakdown of cumulative environmental impact (84.66 g CO_2_-equivalent capita^−1^ day^−1^) of land-locked Czech fish food plate (Table 1) by different groups of aquatic foods. *Subjects of further case study. Minor categories data include (in order of legend): pangasius, tilapia (3.59%), surimi (0.18%), oyster, clam (0.12%), shrimp (0.53%), pike, perch, catfish (0.45%).

**Fig. 2:**
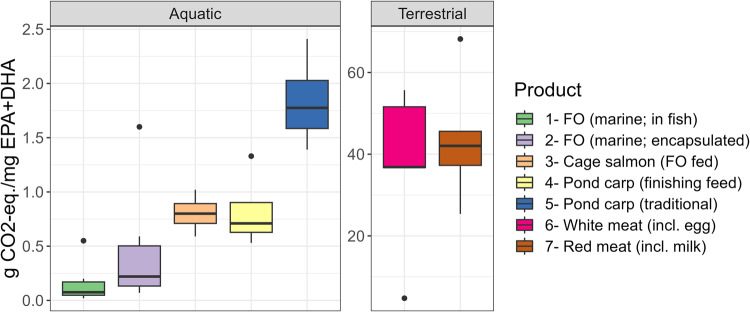
Greenhouse gas emissions of different sourcing options of EPA + DHA for human food basket. Black dots are outliers (clarified in text). The box’s upper bound is the 75th percentile, the lower bound is the 25th percentile, the center line is average, and the whiskers are maxima or minima. Abbreviations: FO fish oil, incl. including.

The estimated cumulative environmental impact of EPA + DHA intake from aquatic products in Czechia (Table 1) is 84.66 g CO_2_-eq. capita^−1^ day^−1^. A breakup of this can be found in Fig. 1. Marine fish, fish oil capsules, and farmed salmonids have the highest contributions, followed by locally farmed carp. The study further examines sustainability and resource use efficiency aspects of EPA + DHA from marine fish, farmed salmon, and pond-farmed carp (Fig. 2; next section).

### GHG emissions associated with increasing EPA + DHA intake from different sources

Terrestrial animal-sourced products have a 10:1 scale higher GHG emission (>30 g CO_2_-eq. mg EPA + DHA^−1^) than aquatic animal-sourced EPA + DHA ( < 2.5 g CO_2_-eq. mg EPA + DHA^−1^) (Fig. 2). As outliers, goat or lamb meat have excess, but chicken egg with a high content of EPA + DHA show least GHG emission (4.74 g CO_2_-eq. mg EPA + DHA^−1^) (Fig. 2).

Among aquatic sources, EPA + DHA within fish oil in wild-captured marine fish shows the least GHG emission. The outlier can be cod (0.55 g CO_2_-eq. mg EPA + DHA^−1^), followed by Alaska pollack (0.20 g CO_2_-eq. mg EPA + DHA^−1^) (Fig. 2). However, as that oil is extracted from rest of the solid biomass, refined, re-manufactured within gelatine-based capsules, and packaged in a way to retain oxidative stability during shelf-life, the GHG emission of encapsulated EPA + DHA become higher (Fig. 2). The outliers could be cod liver oil capsules (1.60 g CO_2_-eq. mg EPA + DHA^−1^) followed by Alaska pollack derived capsules (0.59 g CO_2_-eq. mg EPA + DHA^−1^). These values tend to match or even exceed in-fish EPA + DHA in farmed salmon or farmed carp with finishing feeding (Fig. 2).

**Fig. 3:**
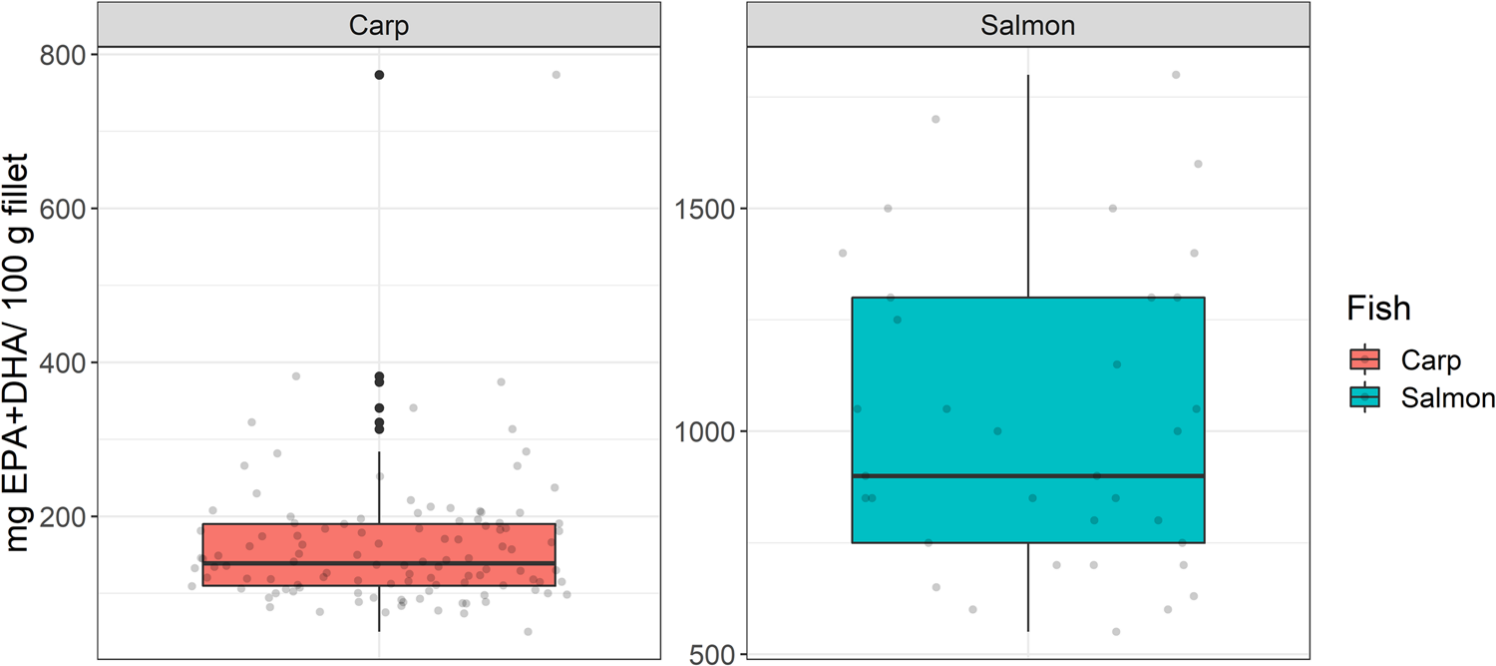
Content of EPA + DHA in semi-intensively farmed common carp from Czech fishponds and intensively farmed salmon of Scottish or Norwegian origin in RAS or cage. Black dots indicate some outliers, achievable by finishing the feeding strategy. The same definition of box-whiskers as in Fig. 2.

Intensively farmed salmon have lower GHG emission per unit mass of EPA + DHA contributed to human food basket than extensively to semi-intensively farmed pond carp (Fig. 2). Large differences presently occur in nutritiousness of farmed salmon (interquartile range, IR: 750–1300 mg EPA + DHA 100 g^−1^ fillet) over traditionally farmed carp (IR: 101–181 mg EPA + DHA 100 g^−1^ fillet) (Fig. 3). Fortifications in pond farmed carp done by finishing feeding strategy (>300 mg EPA + DHA 100 g^−1^ fillet), is necessary to reduce GHG footprint of carp and make it comparable to salmon (Fig. 1). However, a level of 200 mg EPA + DHA 100 g^−1^ carp fillet may be insufficient to achieve environmentally comparable status with salmon (Fig. 1; outlier point). Maintaining a dense EPA + DHA in either farmed salmon (1300 mg EPA + DHA 100 g^−1^) or pond carp (500 mg EPA + DHA 100 g^−1^) with a finishing feeding strategy, the GHG emissions from farmed fish fillets could be as low as 0.59 g CO_2_-eq. (salmon) or 0.53 g CO_2_-eq. (carp); lower than even omega-3 capsules containing 250–300 mg EPA + DHA (Fig. 2).

However, beyond GHG emissions, there are other aspects to consider as well. In the following sections, they are further examined between salmon and carp production models.

### Farm effluent and ecosystem services between carp and salmon

**Fig. 4:**
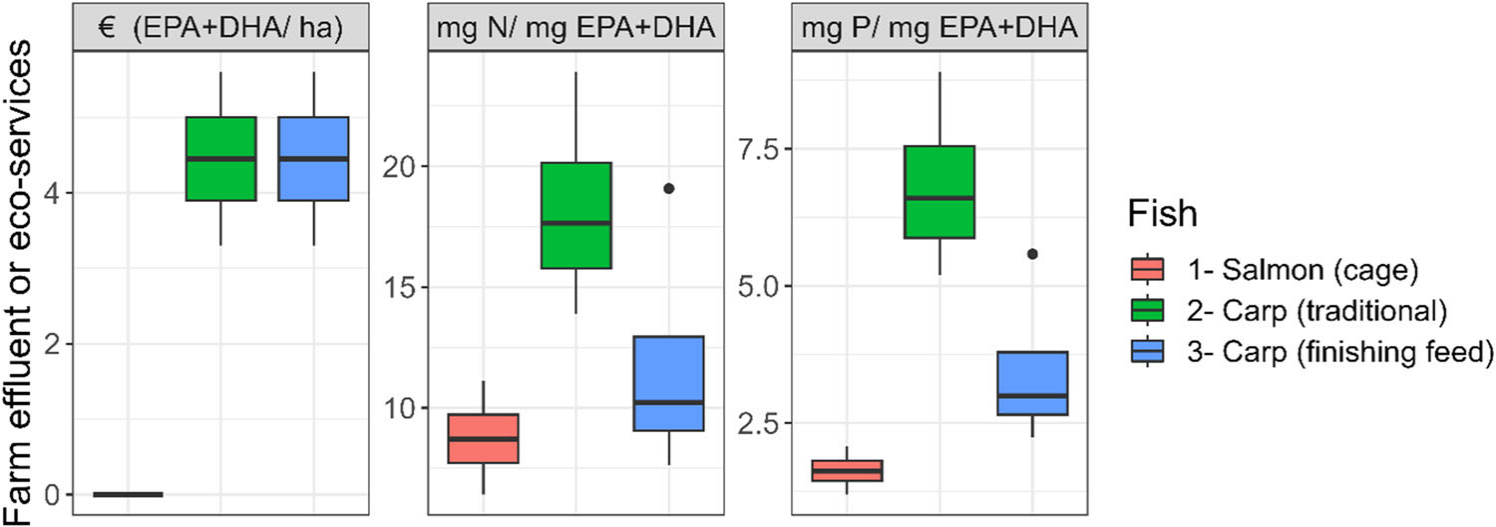
Farm nutrient effluents and ecosystem services per unit mass of EPA + DHA contribution to human food basket by semi-intensive pond carp or intensive cage salmon. The same definition of box-whiskers as in Fig. 2.

The N and P effluents caused per mg of EPA + DHA are lower in salmon than in carp (Fig. 4), although the latter is less intensive and cleaner production-wise. Like GHG emission, the reason can be attributed to less EPA + DHA richness of traditionally farmed carp (Fig. 3). Only when a finishing feeding strategy is used and despite some increased N and P effluents in ponds, the N and P effluents could match the level of salmon (~10 mg N, ~2.5 mg P per mg EPA + DHA). The differences may cease to exist at >300 mg EPA + DHA 100 g^−1^ carp fillet (Fig. 4). However, an improved level of 200 mg EPA + DHA 100 g^−1^ carp fillet may be insufficient to create any meaningful differences beyond the traditional carp (Fig. 4; outlier point). The trends are consistent with that of GHG emissions in the previous section.

However, EPA + DHA obtained through pond-farmed carp may bring some valuable eco-services worth >4 € in EPA + DHA mined per hectare of pond. Even with a finishing feeding scenario, the baseline EPA + DHA contributed to carp via the pond food web would have its contributions, resulting in an intact eco-service profile (Fig. 4). Such positive ecosystem services are not usually recognized for intensive and confined production systems such as salmon (Fig. 4).

### Resource use efficiency between carp and salmon production system

**Fig. 5:**
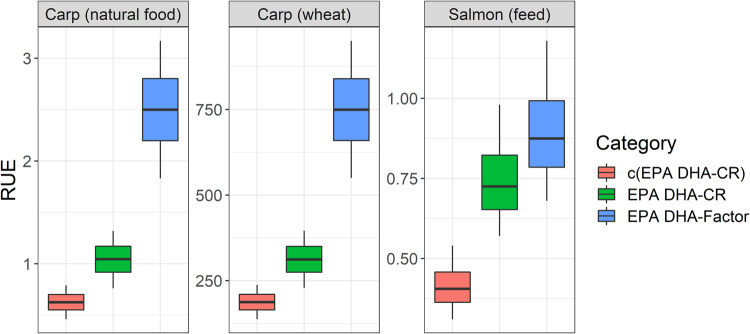
EPA + DHA resource use efficiency (RUE) in pond carp fed on supplementary feed (cereals) or natural food (zooplankton, zoobenthos) and intensively farmed salmon fed on a fish oil-based diet. *Legends:* c(EPA DHA-CR) = ‘consumable’ EPA + DHA conversion ratio. EPA DHA-CR = ‘gross’ EPA + DHA conversion ratio. EPA DHA-Factor = concentration times higher or lower in the flesh compared to food. Comparison on iso-edible yield basis (60%) of salmon and carp—see Method for clarifications. The same definition of box-whiskers as in Fig. 2.

When compared between cage-farmed salmon (fed with FO-based diet) and pond carp fed with cereals, the EPA + DHA conversion ratio of carp far exceeds salmon (Fig. 5). Pond carp can show extraordinary output of EPA + DHA for human food chain than it derived (from human food chain) by use of cereals (Fig. 5). This is because EPA + DHA in carp come from pond food web, not from fed cereals.

So, we provide a comparison between cage-farmed salmon fed on pellets and pond carp fed on zooplankton–zoobenthos. Here too, the EPA + DHA conversion ratio in carp seems higher (gross = 1:1; consumable = 0.6:1) than in farmed salmon (gross = 0.75:1; consumable = 0.4:1) (Fig. 5).

EPA + DHA concentration in carp flesh is higher by a factor of ~2.5 than natural prey items in the pond (~0.06% EPA + DHA as-fed basis). Whereas EPA + DHA concentration in salmon flesh resembles that of feed (1.1% EPA + DHA as-fed basis), salmon EPA DHA-Factor is ~0.90–1.0 (Fig. 5). The mechanisms behind pond carp are further examined in the next section.

### EPA + DHA bioavailability and accumulation from pond diet to carp

#### Bioavailability

The EPA + DHA content in experimental diets was: HIGH (160.1 mg EPA + DHA 100 g^−1^), BALANCED (128 mg EPA + DHA 100 g^−1^), and LOW (60.1 mg EPA + DHA 100 g^−1^). The bioavailability of EPA + DHA from experimental diets was consistently high and as follows: (a) high diet 91%, (b) balanced diet 90%, and (c) low diet 91%.

#### Accumulation pattern

**Fig. 6:**
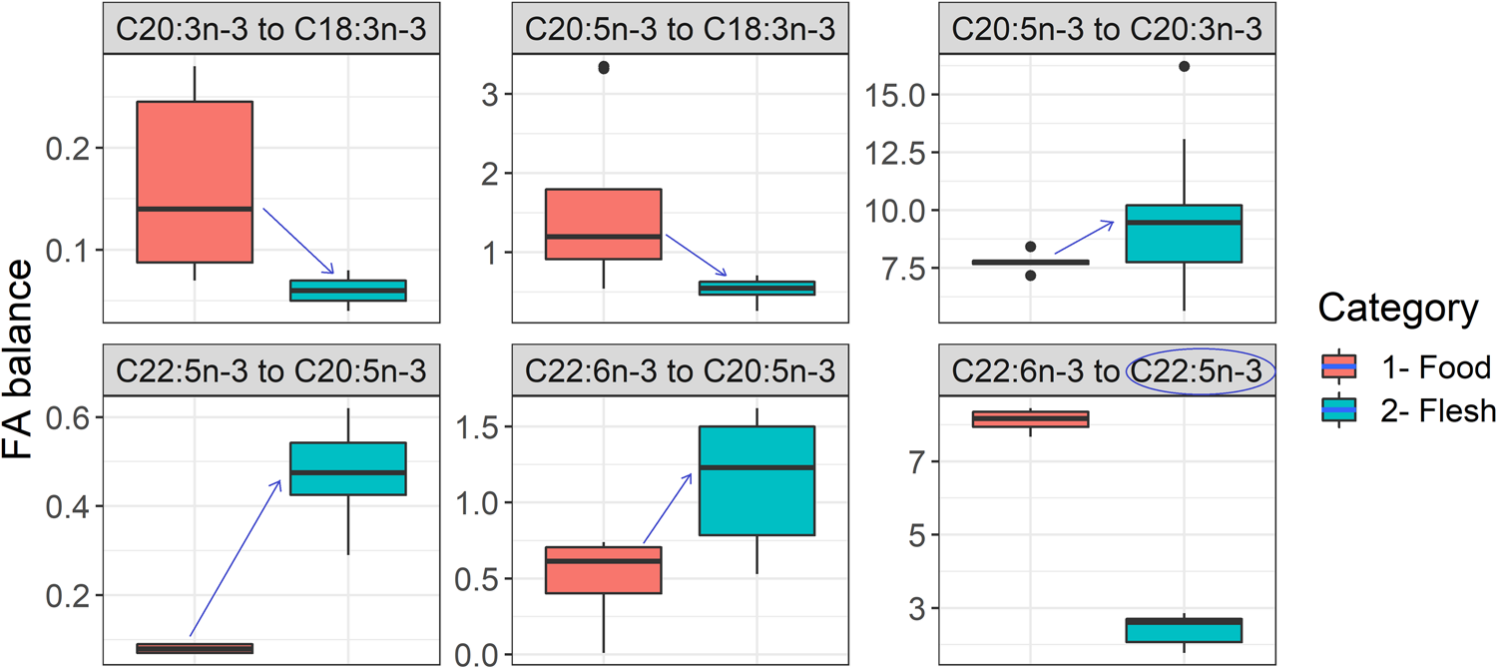
Fatty acid balances (molar-by-molar basis) from food to flesh of common carp in traditional fishponds. The same definition of box-whiskers as in Fig. 2.

First, the upward arrow from food to flesh (Fig. 6) indicates carp accumulate more EPA (numerator) relative to C20:3ω-3 (denominator) and also accumulate more DPA and DHA (numerators) relative to EPA (denominator) than ratios originally existed in food base.

Second, the downward arrow from food to flesh (Fig. 6) indicates carp accumulates a large amount of ALA (denominator) relative to C20:3ω-3 (numerator) or EPA (numerator), opposite to the ratio that exists in food. Likely because C20:3ω-3 gets elongated to EPA, and EPA gets converted to DHA (above points).

Third, in the food base, DHA (numerator) largely exceeds relative to DPA (denominator). But in the flesh, the gap is narrowed, possibly due to the appearance of more DPA (increase in the denominator) (Fig. 6).

## Discussion

Cardiovascular mortality is particularly concerning in CE. Dietary recommendations by the European Food Safety Authority (EFSA) for EPA and DHA based on cardiovascular risk considerations for European adults are between 250 and 500 mg day^−1^ ^1^. Clinical trials in the Czechia^14,15^ revealed that Czechs consume 3–4 times less fish than the EU average. Most CE countries consume at least 2–2.5 times less fish than the EU average. Czech clinical trial participants, who consumed fish meal at least two times per week or fish oil capsules daily, had a mean omega-3 index of 4.10% compared to those who ate fish less than once time a month (omega-3 index < 2.5%)^14^.

The present study supports that cardiovascular disease rates and GHG footprints from ruminant meat intake could be lowered through moderate consumption of aquatic food with low environmental impact^22^. Results in Table 1 suggest that increased consumption of oily and captured marine fish species could easily fulfill the minimum recommended EPA + DHA intake in Czechia. However, going beyond the minimum recommended intake, i.e., >300 mg EPA + DHA day^−1^, may create serious dependency of the landlocked region on marine resources or intensively farmed fish. Results in Fig. 2 suggest increasing EPA + DHA intake in Czechia by keeping the lowest environmental impact would require pond-farmed fish with a finishing feeding strategy (using local circular fish oil; mentioned below). This would also reduce pressures on marine resources^23^ or intensively farmed salmonids with marine resources^8^ or conflicts of fish oil capsule sector with animal feed^24^. Inland fishes are overlooked for EPA + DHA security^25^.

Taking the median values of EPA + DHA content (Fig. 3), the consumption of carp fillets would have to be 156–180 g (traditional) or ≤100 g (finishing feed fed) to satisfy the minimum recommended intake of 250 mg EPA + DHA day^−1^. In comparison, 25–28 g of salmon fillet or one fish oil capsule would satisfy the same amount of EPA + DHA. It corresponds to a clinical trial in Czechia which observed not so improved omega-3 index in participants who were over-reliant on traditionally farmed carp^14^. Usually, a higher serving of pond fish species is needed (371–666 g) to fulfill a weekly intake demand by an adult (=250 mg EPA + DHA × 7 days)^26^. Comparatively, only 121–212 g of intensively farmed rainbow trout, brook trout, or northern whitefish could fulfill the same weekly demand (1750 mg EPA + DHA) of an adult^26^. The annual Czech fish consumption is so low (see introduction) that the above-recommended amounts for pond fish are seldom eaten. Besides, a high intake of saturated fatty acids via terrestrial meat (see “Introduction”) may have confounded no improvement in the omega-3 index of frequent fish eaters^14^. A reduction in saturated fatty acid intake (butter, meat) may have been necessary also.

From a GHG emission perspective, fish oil capsule may not always seem to be the responsible choice relative to appropriately farmed fillets (Fig. 2). Direct consumption of captured marine fish with ‘in-fish’ EPA + DHA may bring lower GHG emission than ‘encapsulated’ EPA + DHA. There is a longer value chain with fish oil capsule manufacturing, packaging, and distribution value chain. That involves secondary processing or transport for oil extraction and oil refining; also, gelatin-based coating manufacture, stabilizers, careful packaging for oxidative stability, etc.^27,28^. Also, LC PUFAs in capsules may be over-oxidized due to the production and storage, attenuating its benefit for human health^29^. Cardiovascular, anti-aging, or cognitive protection by fish is also contributed by fish protein peptides (FPP), vitamins (A, D), and minerals (selenium)^6,7^; not only EPA + DHA. Particularly, the health benefits of consuming FPP, irrespective of marine or freshwater fish, are well established by in-vivo studies; summarized recently^30^. A synergistic health benefit effect of increasing EPA + DHA intake in CE may only be possible if whole-fish or FPP are consumed too. This corresponds to some previous research. Pond-farmed carp have been used as a tool in the secondary prevention of ischemic heart disease in patients following revascularization surgery in the Czechia^31–33^. In 1980s, efforts were made in Germany to treat hypertensive patients with locally farmed carp consumption^25^. These were generally successful when eating fish in two portions a week (200 g per portion) involving both FPP and EPA + DHA intake together.

Results in Fig. 4 suggest a similar-to-lower nitrogen and phosphorus effluent per mg EPA + DHA than salmon may be possible in carp if fillet EPA + DHA exceeds >300 mg 100 g^−1^. Any reduction in salmon fillet EPA + DHA contents could result in more nutrient effluents (per mg EPA+DHA) than carp. Historically decreasing fishmeal and fish oil inclusion have corresponded with reduced ω3 PUFA content in feed and fillets of farmed salmonids^10^. If farmed salmon are not fed with high proportion of EPA + DHA^2^, the resultant EPA + DHA content could drop from ~1300–2700 mg EPA + DHA 100 g fillet^−1^ ^2^ to below 750 mg EPA + DHA 100 g fillet^−1^ in wild salmon which preys upon wild fish^10^. For example, intensively farmed rainbow trout in Czechia have ≥750 mg EPA + DHA 100 g fillet^−1^, whereas from extensive farming they have below 200 mg EPA + DHA 100 g fillet^−1^; close to traditional carp in Czech ponds^26^. The salmonid aquaculture industry has been actively scoping for alternatives to fish oil, such as krill oil, algal oil, transgenic canola, and camelina oil^2,8,34^. This needs to be done with the pond industry too, but with a finishing feeding strategy (mentioned above), as pond carp already derive some LC PUFAs from the pond food web (discussed). “Finishing feeding strategy” is a resource-efficient^35^, nutritious^36^, and value-addition strategy^34^ in aquaculture. Carp in Czechia has a multi-year production cycle (2–3 years). In the final year of market size carp, this strategy could cut GHG emissions of EPA + DHA (Fig. 2).

There have been some efforts to increase EPA + DHA in carps^37–39^. A patented omega-3 carp production model in ponds also exists, producing from 200 mg EPA + DHA 100 g fillet^−1^ up to 351 mg EPA + DHA 100 g fillet^−1^ ^32^. Re-directing local fish processing wastes (going to pet food) and ‘reduction fishery’ harvests from drinking water reservoirs (going to incineration) for extracting ‘circular’ fish oil is a possibility^40^. In Czechia, 87–100 tons of such circular fish oil (>3% EPA + DHA content) is estimated to be extractable (Roy, Mraz, unpublished data). Fish oil at an inclusion rate of 9% fed to ponds through a finishing feeding strategy for 110 days could alleviate EPA + DHA levels in carp fillets up to ~550 mg 100 g fillet^−1^ ^41^. Another finishing feeding experiment in ponds showed carp fed at 7% fish oil inclusion for 30 days prior to harvest had EPA + DHA content ~278–338 mg 100 g^−1^ fillet; 13.9–16.9 g EPA + DHA g^−1^ dry muscle^42^. When pond carp was fed with a fish oil-based diet (13% inclusion) for a fuller length of the season (≥210 days), the result was even higher^43^. Considering an average of both polar and non-polar lipids, dorsal and ventral muscles, and 80% moisture content of muscle, the average EPA + DHA content could reach 960.2 mg 100 g^−1^ fillet^43^.

Conforming to findings from previous analyses^3,4^, results in Fig. 5 suggest pond carp system could be highly resource efficient in farming EPA + DHA. Farmed salmonids have a low omega-out-omega-in conversion ratio (ωCR ~ 0.4), while farmed carp are reported to have ωCR ~ 50:1^3,4^. For limited circular and freshwater FO available in Czechia, pond fish with a high RUE could be economic candidates for short-finishing feeding (30–90 days). A dilution model for pond-farmed carp is already available, which can assist farms in predicting carp fillet EPA + DHA under fish oil-based finishing feeds and different durations of feeding^41^. In European fishponds, the advantages lie in lower stocking density^44^, contributions of EPA + DHA from natural food web^45^, and endogenous capacities of pond fish (below). Besides finishing feeding, polyculture could be resource-efficient. For example, in Czech ponds, EPA + DHA content in wels catfish, silver carp, or tench could be naturally higher than common carp^26^. But not enough to balance out sustainability aspects (Figs. 2 and 4) or complement intensively farmed salmonids^26^. However, the recognition of ecosystem service of fishponds, including any farmed nutrients out of them, likely out-compete other aquaculture models^46,47^. Pond systems may counter-balance the need to expand or any negative ecosystem service of farmed salmonids^48,49^.

In ponds, zooplankton and benthic macroinvertebrates almost entirely contribute to EPA + DHA in carp muscles^45^. They are trophic transfer agents of fatty acids from the base of the fishpond food web (i.e., algae, bacterioplankton) to fish biomass^50,51^. Our experimental results show their bioavailability is high. However, in a recent study^52^, it was shown that EPA + DHA digestibility from lyophilized zooplankton can deteriorate in carp fed with high acid detergent fiber levels or specific non-starch polysaccharides. So, the composition of finishing feed may be starchy (wheat or triticale-based) to ensure high fatty acids bioavailability (from fish oil or zooplankton) as well as lipid sparing. In ponds, a low stocking density and artificially increasing submerged beds for zooplankton–zoobenthos–periphyton^53^ could increase EPA + DHA output as well. Pond carp may defend natural food-derived n-3 PUFAs during starvation and purging^54^. If some finishing feeding strategy during mid-summer to autumn (≤90 days) with fish oil (≥7% inclusion), high starch (>40%), and low protein (20–25%; pea, wheat combination) is fed to fishponds, the de novo lipogenesis phenomenon occurring at the end-of-season (before harvests), priming carp for ‘potential’ overwintering^55,56^, could be exploited. Usually, the period from the 3rd week of July to the 2nd week of September increases fat deposition in carp occurring in regional fishponds^57^. During this period, protein accretion or growth deteriorates^55^. An accumulation of EPA + DHA in non-growing biomass may be targeted.

Findings in Fig. 6 support recent findings in regional fishponds that DHA in pond carp may be generated endogenously via bioconversion from EPA^45^. Because food based in freshwater ponds or lakes can be selectively rich in ALA or EPA but generally poor in DHA^45,50,58^. In our dataset, copepods (ALA: 0.467%, EPA: 0.513%, DHA 0.366% of DM) seemed most rich, followed by cladocerans (ALA: 0.391%, EPA: 0.215%, DHA: 0.087% of DM) and chironomids (ALA: 0.110%, EPA: 0.365%, DHA: 0.003% of DM). Despite both DHA: EPA and DPA: EPA ratios in the flesh being greater than in food, the DHA: DPA ratio could not be higher in the flesh than food (Fig. 6). DHA biosynthesis may be putative in pond carp, whether via the Sprecher pathway needs to be validated.

Similarly, EPA biosynthesis in carp is recognized via the Δ6Δ5 pathway; see supplementary information in Monroig and colleagues^59^. The amount of [1-^14^C]18:3n-3 converted to pentaene products (*i.e*., total radioactivity recovered as 20:5*n*−3 and 22:5*n*−3) was ~50–66% in carp cell lines^60^. In another study, 51–61% of the radioactivity of radiolabelled [1-^14^C]18:3*n*−3 could be recovered in an average of Δ6 desaturase, C_18–20_ elongase, and Δ5 desaturase products^61^. Thus, up to half of a surplus ALA gradient (relative to a lower EPA gradient) may be triggered toward EPA biosynthesis. For example, a blend of linseed oil and ‘freshwater’ fish oil in finishing feeds. In the same study^61^, [1-^14^C]18:3*n*−3 also got elongated and desaturated to C20:3*n*−3 and C20:4*n*−3, respectively. Δ8Δ5 pathway for EPA biosynthesis is also believed to be a possibility in cyprinids, as in *Barbynomus gonionotus* reviewed^59^. The transgenic yeast cell model transformed with the zebrafish fatty acyl desaturases (expressing bi-functional Δ6 and Δ5 desaturase) also expressed limited Δ8 desaturase activity converting 1.5% of C20:3*n*−3 to C20:4*n*−3^62^. Results of Fig. 6 show in some cases, a higher EPA to C20:3*n*−3 ratio in the flesh is possible than originally present in food. It opens speculation about whether Δ8Δ5 pathway is rudimentarily active in pond carp—the subject of future investigation. Because zooplankton–zoobenthos seem to provide the pre-cursor, C20:3*n*−3: cladocerans (0.066% of DM), copepods (0.028% of DM), and chironomids (0.031% of DM). Nonetheless, pond carp do upregulate certain desaturases and elongases either as cold-response^63–65^ (CE ponds are temperate) or to restore fatty acid balances in food^45^. If carp with high enzyme activity in FA elongation and desaturation are bred^66,67^, the pond sector may become a valuable producer of EPA + DHA. Research priorities are needed in this regard.

The authors conclude overall fish consumption needs to be increased in CE (e.g., Czechia) to fix an ailing EPA + DHA security in the region. Although marine fish or intensively farmed are the mainstay of EPA + DHA security, the fish food platter needs to be designed with inland-marine fish combinations. To allow EPA + DHA security with minimum pressure on the environment or resources. To do that, it is necessary to modernize traditional pond farming in CE. To produce multiple pond fish that would aid in future EPA + DHA security (increased fish consumption), with lowered footprints, increased ecosystem services, and complementing salmonids/ captured marine fish. The pond sector has potential to be a net producer of EPA + DHA for human food basket but require advancements such as circular finishing feeding and future research on bioaccumulation pathways to do it more effectively.

## Methods

### Characterization of EPA + DHA source and supply in land-locked CE

We took Czechia (a developed economy) as a representative of the predominantly land-locked CE region. Based on fish food balances (*i.e*., import, export, production, consumption mass balances) of Czechia^68^, surveys of fish consumption, inventory of fat and EPA + DHA content commonly eaten fish products in Czechia (Jan Mraz, unpublished results, 2010), we estimated per capita fish consumption patterns and the resultant EPA + DHA supply (representative values). The Czech fish food plate was further refined by considering fish oil capsule consumption in the country. In the absence of conclusive data on fish capsule consumption in the country, results from a previous clinical trial in the Czechia were taken, which showed 10% of the subpopulation take omega-3 supplements (fish oil capsules) regularly^14^. Czech population statistics (~10.5 million; https://www.czso.cz/csu/czso/population), per capita annual fish consumption (6 kg), per capita locally produced pond fish consumption (1 kg) were taken from the Czech Statistical Office (CZSO) estimate^17,18,69^.

### Quantification of carp, salmon, fish oil capsules, and other reference values

#### EPA + DHA content in extensively to semi-intensively farmed carp in CE fishponds

As a representative of pond-farmed fish (for local human food baskets) in land-locked CE, common carp (*Cyprinus carpio*) typically farmed in Czech fishponds was selected. Czech pond-farmed carp had a comprehensive dataset necessary for detailed evaluation.

The range of EPA (C20:5-ω3) and DHA (C22:6-ω3) content of traditionally farmed *C. carpio* (common carp) in Czech and German fishponds fed with cereals only (as supplementary feeding) was compiled from multiple datasets; covering fillets from over 100 carps analyzed in the same laboratory using a standard set of methodologies involving lipid extraction, fatty acids derivatization, and fatty acid determination in GC-FID over last 5 years^26,31,39,54,70–72^, including unpublished author results. A detailed methodology of fatty acid analyses performed in the laboratory is explained in previous studies^54,71,72^. We considered well-fed and locally farmed carp with well above 3% body lipid reserves (wild carp are leaner;^26^). The edible yield of up to 60%, comprising (45% fillet + 5% separated meat from filleted carcass + 10% edible organs and contents of head)^40^ of regional market-sized common carp (>2 kg) was used to upscale edible fatty acid supply per kg of farmed carp biomass from fishponds. The iso-edible yield basis (60%) comparisons of salmon and carp values were based on typical Czech or CE consumption patterns. Typically, salmon (imported) are mostly procured on a fillet basis from supermarkets, while carp (produced and sold locally) are traditionally eaten in the region both in the form of fillets and soups, meatballs, or sausages (low-cost value-added products from leftovers after filleting). With these assumptions, the study was designed for the land-locked CE. All calculations in the following sections were made from the 25th percentile, mean, median, and 75th percentile values of EPA + DHA.

#### EPA + DHA content in intensively farmed salmon in flow-through systems

Due to comparable EPA + DHA content with other captured marine fish, similarities with intensively farmed freshwater salmonids (trout), and comprehensive data availability on farmed salmon, we selected data on farmed salmon fillets (Scottish or Norwegian origin).

EPA + DHA contents per 100 g farmed salmon fillets (of Norwegian and Scottish farm origin) were taken from published observations^2,10^. Farmed salmon’s edible yield (mainly fillet) ~60%^73,74^ was used to upscale edible fatty acid supply per kg of farmed salmon biomass.

#### EPA + DHA content in fish oil capsules and GHG footprint

We considered the usual range of EPA + DHA content (200–300 mg; median 250 mg) in most affordable fish oil (anchovy, sardine, mackerel, herring) capsules in the Czechia, and the recommended serving (2 capsules day^−1^; 1000–1200 mg fish oil capsule^−1^)^75^. There is inconclusive data on fish capsule consumption in Czechia. So results from a previous clinical trial in the Czechia were assumed, which observed that 10% of the clinical trial participants in the subpopulation take omega-3 supplements (2 capsules or 2 g fish oil day^−1^) regularly^14^. So, it was extrapolated over the entire population as 0.2 g fish oil (from capsule) consumption per capita per day.

First, the GHG footprint per unit of EPA + DHA in captured marine fish was taken from supplementary data in a previous study^76^. GHG emission data per 0.5 g EPA + DHA provided by the authors^76^ from wild captured salmon (33.31 g CO_2_-eq.), herring (12.29 g CO_2_-eq.), mackerel (18.78 g CO_2_-eq.), tuna (41.17 g CO_2_-eq.), cod (273.70 g CO_2_-eq.), and pollack (101.30 g CO_2_-eq.). Second, the GHG emission of extracting and refining such oil and encapsulating it by capsule manufacturing process was accounted for; using a case study on captured krill biomass converted to omega-3 capsules^28^. The relative change in GHG footprint is by a factor of ≈2.93; from krill biomass/meal at primary processing (5.4 g CO_2_-eq. g^−1^) to extracted and refined krill oil in gelatine-based omega-3 capsulates (0.95 kg CO_2_-eq. per 60 capsules containing total 60 g oil; equivalent to 15.83 g CO_2_-eq. g^−1^)^28^. Finally, the factor (2.93) was multiplied by the above values for different marine fish. This gave the GHG footprint of marine EPA + DHA encapsulated in gelatine-based coating and intended for direct human consumption. The average of in-capsule values or in-fish values was used for quantifying EPA + DHA-related emissions derived from omega-3 capsules or captured marine fishes (fillet or canned or smoked) in the Czech fish food plate (Table 1).

#### GHG footprint (at slaughter) of carp, salmon, and terrestrial meat

GHG footprint data (1.59 kiloton CO_2_-eq. per kiloton carp produced) of Eastern European and Russian pond-farmed carp was taken from a peer-reviewed FAO assessment^77,78^. The data is representative of semi-commercial extruded or farm-made pressed pellets (intended for semi-intensive ponds) made from a mixture of local ingredients for *C. carpio* aquaculture in Russia and Eastern Europe. If traditional wheat (368 g CO_2_ eq. kg^−1^; https://www.feedtables.com/content/wheat-soft) application in regional semi-intensive fishponds is considered (FCR 2–2.5), GHG footprint could amount to 0.74–0.92 kiloton CO_2_-eq. per kiloton carp produced. Therefore, we used the higher value (1.59 kiloton CO_2_-eq. per kiloton carp produced) to account for an advanced farming method that may use pellets or low-cost finishing feed prepared from local and waste-stream ingredients fed to ponds.

The GHG footprint data of farmed salmon was taken from a Norwegian industrial assessment^79^. The median value (4.57 kiloton CO_2_-eq. per kiloton salmon produced) calculated from the reported range (2.9–6.25 kiloton CO_2_-eq. per kiloton salmon produced) was used for calculations; relying completely on commercial and extruded pellets with refined ingredients.

As a reference value to fish oil or whole farmed fish, data on two categories of terrestrial animal were considered: (a) white meat comprising chicken, turkey, and duck including eggs; and (b) red meat comprising beef, veal, pork, and lamb including milk. The minimum net GHG emission data reported for EU poultry meat (5 kg CO_2_-eq. kg meat^−1^), chicken egg (2.8 kg CO_2_-eq. kg egg^−1^), pork (7 kg CO_2_-eq. kg meat^−1^), goat/ lamb (19 kg CO_2_-eq. kg meat^−1^), and cow milk (1.3 kg CO_2_-eq. kg milk^−1^) were taken from a previous EU-27 assessment^80^. The EPA + DHA contents per 100 g of selected products were taken from a standard database (AUSNUT 2011–2013 food nutrient database): beef (35.77 mg), lamb (27.86 mg), veal (56.34 mg), ham (16.65 mg), pork (7.28 mg), chicken (9.69–13.61 mg), turkey (13.57 mg), duck (8.98 mg), chicken egg (59.12 mg), and cow milk (5.12 mg).

#### Farm nutrient effluents of carp and salmon production

For farm nutrient effluents, combined fecal, non-fecal, and uneaten feed loadings of nitrogen (N) and phosphorus (P) were considered. Data from a previous estimate on Central European carp ponds^44^ was taken. Out of the reported range (15.8–29.9 kg N and 5.9–7.5 kg P ton^−1^ of carp produced), the minimum values (15.8 kg N, 5.9 kg P ton^−1^) were used for traditional and non-finishing feed-based production estimates. A hypothesized (advanced) production method involving four levels of finishing feeding strategies in ponds was considered: (a) patented omega-3 carp (from 200 to 350 mg EPA + DHA 100 g^−1^)^32,33^ and (b) fish oil-based feed (from 400 mg to >500 mg EPA + DHA 100 g^−1^)^41–43^. For finishing feeding strategies in ponds, a higher farm nutrient effluent was considered. The median value (22.9 kg N and 6.7 kg P ton^−1^) of the above range was used. Data from a previous estimate on Norwegian cage salmon farm^81^ was taken as follows: 50 kg N and 9.3 kg P ton^−1^ salmon produced.

#### Ecosystem services valuation of carp and salmon production

Unlike pond carp (or semi-intensive production methods), ecosystem services valuation of intensively farmed salmon in flow-through systems has not been recognized or quantified so far^82,83^. However, there could be ecosystem services associated with EPA + DHA mining (farming) from CE fishponds utilizing pond food web for extensive to semi-intensive production methods^44^.

For the calculation, present carp production data (~20,000 tons per annum from 44,000 ha Czech fishponds; *data*: CZ-Ryby), ecosystem and (aqua)-cultural services of Czech fishponds (∼2375 € ha^−1^), average annual yield (449.4 kg ha^−1^)^44^, total edible yield from carp (up to 60%) and average moisture content (~70%; no value)^40^ were considered. The valuation associated with 1 mg of edible dry matter, including oil (~0.000011 EUR), was upscaled (multiplied) with a consumable yield of EPA + DHA (659.8–1140.6 mg consumable EPA + DHA per kg carp yield) produced with fish per hectare of Czech fishponds (296513.1–512604.5 mg EPA + DHA per hectare yield).

#### EPA + DHA resource use efficiency (RUE) for carp and salmon models

Three indices *viz*. EPA + DHA conversion ratio at farm level (EPA DHA-CR), consumable EPA + DHA conversion ratio at fork level [c(EPA DHA)-CR], and EPA + DHA concentration factor (EPA DHA-Factor) were used as measures of EPA + DHA resource use efficiency (RUE) of farmed species or production systems, from a perspective of the human food basket. “EPA DHA-CR” on supplementary feed (wheat) and natural food base were calculated separately. Whole-body EPA + DHA content (1099.7–1901.1 mg kg^−1^) was used as a numerator, and EPA + DHA content of either wheat (≤0.2 mg 100 g^−1^ grain) or natural food (≥60 mg 100 g^−1^ live weight/ biomass) multiplied by their relative feeding coefficient (RFC) of original matter (wheat RFC_drymatter_ 2; natural food RFC_wetweight_ 2.4) was used as the denominator. Values were taken from a previous analysis; ‘natural food’ value includes an average of cyclops, daphnia, and chironomid larvae^55^. “c(EPA DHA)-CR” was calculated by multiplying the numerator with edible yield coefficient (0.60)^40^, while the denominator remained same. “EPA DHA-Factor” was calculated by EPA + DHA concentration in fillet as the numerator and EPA + DHA concentration in wheat or natural food as the denominator.

“EPA DHA-CR,” “c(EPA DHA)-CR,” and “EPA DHA-Factor” of salmon were calculated in the same manner as described previously for carp. The difference was in whole-body EPA + DHA content (7500–13000 mg kg^−1^), average EPA + DHA content in feed (1.1%) multiplied with feed conversion ratio (FCR 1.2). The feed EPA + DHA content (1.1%) was averaged from two commercial diets from Aller Aqua and Skretting, corresponding to an intermediate dietary level tested on Atlantic salmon^84^. The edible yield coefficient was 0.6^73,74^.

#### EPA + DHA bioavailability and accumulation from pond diet to carp

All procedures performed in this study involving *C. carpio* were in accordance with the ethical standards approved by the institutional ethics committee regulated by the ministry MSMT CR (certificate no. MSMT-8857/2022-5). Authors team (PD, ZM) have obtained a license for welfare and prevention of cruelty against animals as required by Czech law [section 15e (1), Act no. 246/1992 Coll.].

#### Experiment I: EPA + DHA digestibility from natural food

Three pond-mimicked diets (‘HIGH’ for beginning season, ‘BALANCED’ for mid-season, ‘LOW’ for end-season; detailed in previous study^55^ were mimicked and tested in our 12-tank (120 L tank^−1^) laboratory Guelph-RAS system maintained at optimum feeding conditions (19–21 °C, >4 mg L^−1^ DO, 6.8–7.3 pH). Experimental diets were prepared to combine lyophilized natural prey (freshwater copepods: cladocerans: chironomids @ 1:1:1) of *C. carpio* (common carp) with wheat (common supplementary feed) and 10 g yttrium oxide (inert digestibility tracer) added to 1000 g dry feed mix. Carp stock per tank was adjusted to 4 kg with a mixed cohort (2+ years, 250–400 g) to represent individual variabilities in pond fish stock. ‘HIGH’ diet (80% wheat + 20% natural prey dry matter), ‘BALANCED’ diet (87% wheat + 13% natural prey dry matter), and ‘LOW’ diet (95% wheat + 5% natural prey dry matter) were daily fed to carp stock at 2% of biomass per day, in two divided doses (8:00, 14:00). One unit mass of lyophilized natural prey (freshwater cladocerans, copepods, and chironomids) dry matter roughly corresponds to eight-unit natural prey biomass in original matter (wet weight). The feeding trial continued for 7 weeks. All groups were tested in triplicate. Wheat had no EPA + DHA. Lyophilized zooplankton and zoobenthos were the only sources of EPA + DHA in the diet. Feces were collected, lyophilized using established methodology, and subjected to fatty acids and yttrium analysis relative to their contents in feed. Using standard formula^85^, the apparent diet-level digestibility coefficient (ADC) of EPA + DHA was calculated. Detailed formula, formulation, and composition of three pond-mimicked diets is open-access in a previous study^55^.

#### Experiment II: Fatty acids balance from food to flesh

Fillets from market-sized *C. carpio* (scaly or scaleless; >2.5–3 kg) harvested in autumn (September–October) from fishponds in Vodňany for Christmas sale were sampled for 3 years (2020–2022). Altogether fillets from 24 carp, coming from the traditional production method (ponds supplemented with wheat or triticale), were used for fatty acids quantification. All carp originated from a common source (ponds in Vodňany, Czech Republic, under faculty), having similar ontogeny (age 3+), with a narrow range of stocking density (300–350 kg ha^−1^) and feeding regime (feeding coefficient ~2 kg per kg expected yield of 750–800 kg ha^−1^; May 5% of total dose, June 15%, July 30%, August 35%, and September 15%). Also, fillets were sampled prior to the purging process to avoid disturbances in fatty acid ratio changes^54^.

The fatty acid (FA) quantification was done following pre-established protocols^86^. Data were obtained on fatty acids profile (FA % of total fatty acids). FA ratios (on a molar-by-molar basis) were then computed. Relative appearances or disappearances of FAs, from food to flesh, by tracing sequential changes in FA ratios is a qualitative and surrogate method to explore any putative EPA and DHA biosynthesis^87^. The approach has limitations because ratios can change due to selective accumulation or catabolism of FAs too, besides their bioconversion. Therefore, we used FA ratios to investigate the general accumulation pattern of EPA and DHA and discussed them against literature evidence.

FA ratio(s) towards EPA and DHA accumulation were targeted and retrospectively evaluated based on EPA and DHA biosynthesis pathway(s) validated for carp species models. Usually, carp species are considered to have an efficient bio-machinery^2,59,67^. Also, some EPA + DHA biosynthesis has been confirmed in regional carp ponds using a stable-isotopes approach^45^. So, we calculated six FA ratios in ‘food’ and ‘flesh’: (i) “C20:3ω-3 to C18:3ω-3”; (ii) “C20:5ω-3 to C18:3ω-3”; (iii) “C20:5ω-3 to C20:3ω-3”; (iv) “C22:5ω-3 to C20:5ω-3”; (v) “C22:6ω-3 to C20:5ω-3”; (vi) “C22:6ω-3 to C22:5ω-3”. Then the direction of change from food to flesh was visualized.

It is generally known from previous gut content analysis that the average volume of cereal grain and natural food (only zooplankton, zoobenthos; algae are too small to be filterable by >2.5 kg carp) in carp gut in regional fishponds tend to be close to 1:1 (median over full vegetative season and across fed-unfed sites of ponds)^88^. Therefore, for the ‘food’ category data, the median FA ratio in wheat and natural food was used. For natural food data, the average of FA ratios in freshwater copepod, cladocerans, chironomids, and a generic zooplankton mixture was used (laboratory data) to be representative.

### Reporting summary

Further information on research design is available in the Nature Research Reporting Summary linked to this article.

### Supplementary information

REPORTING SUMMARY

## Data Availability

All data have been made available at Mendeley Data, 10.17632/fy6hxrgw9t.1.
